# Orthobiologics and Peptide Therapy for Central Nervous System Repair in Neurodegenerative Conditions

**DOI:** 10.3390/cells14231853

**Published:** 2025-11-25

**Authors:** Cézar Augusto Alves de Oliveira, Bernardo Scaldini Oliveira, Amanda Scaldini Oliveira, Rafael Duarte de Souza Loduca, Carlos Roberto Massella Junior, Gabriel Silva Santos

**Affiliations:** 1 Neurosurgery, Bioquest Clinic, São Paulo 04508-030, SP, Brazil; 2 Orthopedics, Regeneris Clinic, São Paulo 01404-001, SP, Brazil; bescaldini@hotmail.com; 3 Nutrology, Regeneris Clinic, São Paulo 01404-001, SP, Brazil; ascaldini@hotmail.com; 4 Neurosurgery, Tratar Institute, São Paulo 05426-100, SP, Brazil; loduca65@hotmail.com (R.D.d.S.L.);; 5 Neurosurgery, Military Area Hospital of São Paulo (HMASP), São Paulo 01551-010, SP, Brazil; 6 Brazilian Institute of Regenerative Medicine (BIRM), Indaiatuba 13334-170, SP, Brazil

**Keywords:** Alzheimer’s disease, Parkinson’s disease, orthobiologics, exosomes, peptides, regenerative neurology

## Abstract

**Highlights:**

**What are the main findings?**

- Orthobiologic derivatives and peptide therapeutics converge on shared neurodegenerative pathways, promoting mitochondrial resilience, glial reprogramming, and synaptic repair.
- Intranasal delivery emerges as a practical, minimally invasive route, capable of bypassing the blood–brain barrier while maintaining biological potency.

**What are the implication of the main finding?**

- Combining orthobiologics with peptide-based therapies offers a mechanistically complementary strategy that goes beyond symptomatic relief toward true circuit preservation.
- These insights provide a translational framework for future biomarker-guided and delivery-optimized clinical trials in Alzheimer’s and Parkinson’s disease.

**Abstract:**

Alzheimer’s disease and Parkinson’s disease remain the most prevalent neurodegenerative disorders associated with aging and continue to lack curative treatments. Their pathophysiology is often multifaceted, encompassing protein aggregation, mitochondrial dysfunction, chronic neuroinflammation, synaptic degeneration, and vascular compromise. This complex landscape reduces the effectiveness of single-target pharmacological agents and underscores the need for therapies capable of acting across multiple axes. Orthobiologics and peptide-based strategies exemplify this approach. Autologous cellular alternatives such as platelet-rich plasma, bone marrow aspirates, mesenchymal stromal cell derivatives, and extracellular vesicles deliver paracrine signals that can reprogram glia, preserve mitochondrial function, and promote synaptic and vascular repair. Peptide therapeutics, including glucagon-like peptide-1 receptor agonists and novel sequences targeting protein aggregation or mitochondrial pathways, provide complementary precision by engaging defined receptors and intracellular cascades. Together, these modalities converge on mechanisms central to circuit preservation rather than symptomatic relief alone. Preclinical studies across Alzheimer’s and Parkinson’s disease demonstrate consistent neuroprotective and functional benefits, and early human trials support feasibility and safety. The translational path forward requires standardized preparation, biomarker integration, optimized delivery routes such as intranasal administration, and regulatory frameworks adapted to biologic therapies. This review synthesizes current evidence on orthobiologics and peptides in neurodegeneration, outlines safety and translational considerations, and highlights future directions, including rational combinations and biomarker-driven trials. By uniting the broad signaling capacity of orthobiologics with the precision of peptides, neurology can move beyond symptomatic care toward regenerative strategies that aim to preserve neural circuits and improve long-term outcomes in Alzheimer’s disease and Parkinson’s disease.

## 1. Introduction

Alzheimer’s disease (AD) and Parkinson’s disease (PD) together represent the most common neurodegenerative disorders associated with aging, and they remain without curative therapies despite decades of intensive investigation [1]. Both conditions impose a massive burden on patients, families, and health systems, with incidence rising steadily in parallel with global aging [1,2,3]. Current strategies are largely symptomatic and, at best, modestly disease-modifying, aiming to slow or halt disease progression, leaving a major therapeutic gap [2]. The limitations of conventional pharmacology have stimulated interest in biological therapies that can modulate disease processes more broadly, targeting inflammation, mitochondrial resilience, synaptic stability, and circuit plasticity. Within this context, orthobiologics and peptide-based approaches emerge as promising candidates because they act not through a single molecular blockade but through pleiotropic signaling mechanisms capable of addressing the complex biology of neurodegeneration [4,5].

**Figure 1:**
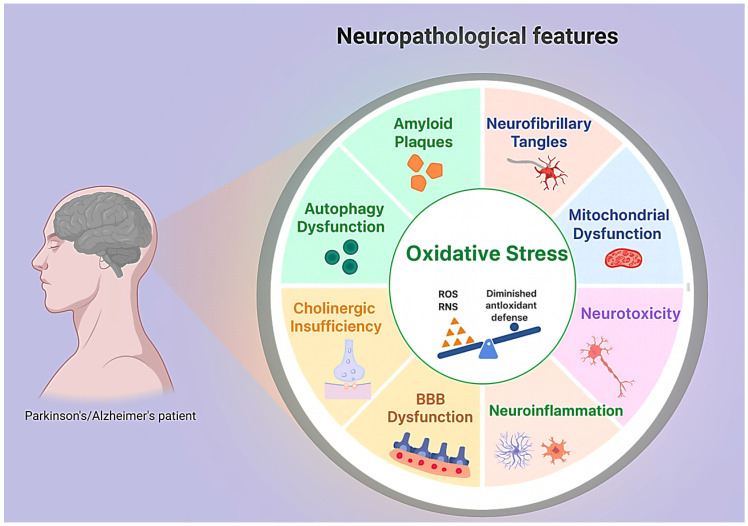
Key cellular and molecular dysfunctions in neurodegeneration.

The two diseases share multiple convergent mechanisms beyond their chronic, progressive nature (Figure 1). AD is driven by the accumulation of amyloid-beta (Aβ) and hyperphosphorylated tau protein, while PD is defined by alpha-synuclein (αSyn) pathology and degeneration of dopaminergic neurons [6,7]. Yet beyond these protein signatures lies a convergence on deeper cellular dysfunctions that include impaired proteostasis, chronic neuroinflammation, disrupted mitochondrial function, synaptic degeneration, and ultimately, the collapse of neural circuits [8,9]. These processes interact in a vicious cycle, where protein aggregation fuels oxidative stress, mitochondrial deficits exacerbate inflammation, and glial activation perpetuates synaptic injury [10,11]. This shared landscape provides a fertile ground for therapies that do not need to eliminate a single pathological protein but can instead rebalance cellular communication, metabolism, and immune responses.

Neuropathological features implicated in Alzheimer’s and Parkinson’s disease. Beyond the characteristic protein aggregates of amyloid plaques, neurofibrillary tangles, and α-synuclein inclusions, both disorders share multiple cellular dysfunctions that converge to drive progressive neurodegeneration. Oxidative stress represents a central hub, amplifying mitochondrial dysfunction, neuroinflammation, and neurotoxicity, while diminished antioxidant defenses exacerbate damage. Disrupted proteostasis, including autophagy impairment, facilitates the accumulation of misfolded proteins. Blood–brain barrier compromise and cholinergic insufficiency further contribute to circuit instability and cognitive decline. The interconnected nature of these processes creates a self-reinforcing cycle that accelerates neuronal injury and loss, highlighting the need for pleiotropic therapeutic strategies such as orthobiologics and peptides that target multiple axes simultaneously.

Orthobiologics represent an especially appealing category for this purpose. Derived from autologous or minimally manipulated tissues such as bone marrow aspirate (BMA), bone marrow concentrate (BMAC), platelet-rich plasma (PRP), or purified extracellular vesicles, these products are already in use in musculoskeletal and regenerative medicine [5]. Their primary action is increasingly understood to lie in paracrine signaling, with growth factors, cytokines, and vesicular cargo modulating local immune tone, stimulating angiogenesis, and enhancing cellular survival [5]. In the central nervous system, these same principles could be harnessed to dampen glial reactivity, improve neuronal resilience, and promote synaptic repair. Mesenchymal stromal cell (MSC) secretomes and their extracellular vesicles have demonstrated the ability to reduce amyloid burden, restore mitochondrial enzymes, and normalize synaptic proteins in preclinical models of AD [12,13,14,15]. In PD models, vesicle-based interventions have shown protection of dopaminergic neurons and improved motor outcomes [16,17,18]. These signals, while preliminary, are compelling because they reflect not isolated effects but rather coordinated shifts in inflammatory and metabolic pathways, considering the complex nature of neurodegenerative disorders.

**Figure 2:**
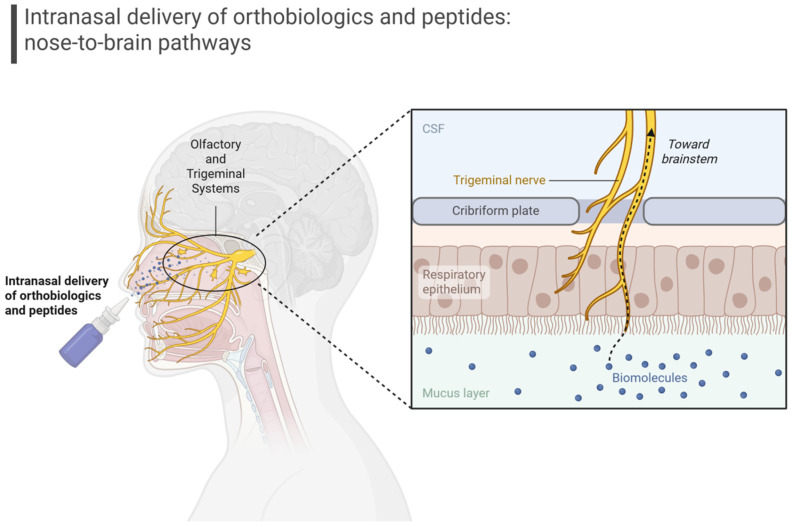
Schematic representation of intranasal/nebulized delivery bypassing the blood–brain barrier via olfactory and trigeminal pathways.

Delivery remains one of the central challenges in bringing orthobiologics into neurology. The blood–brain barrier (BBB) effectively excludes most large molecules and cells from reaching parenchymal targets, making systemic administration insufficient for many interventional strategies [19]. However, intranasal delivery (Figure 2) has emerged as an attractive solution because it can bypass the BBB via direct neural pathways, such as those from the olfactory and trigeminal systems, while avoiding systemic dilution [19].

Schematic representation of intranasal/nebulized delivery bypassing the blood–brain barrier via olfactory and trigeminal pathways. This route provides direct access to the central nervous system and supports translational strategies for orthobiologics and peptide-based therapies in Alzheimer’s and Parkinson’s disease.

Previous investigations have demonstrated that intranasal administration of extracellular vesicles, secretome, and peptides can achieve brain penetration and functional rescue in models of both AD and PD [20,21,22,23,24,25]. Other approaches, such as intrathecal or intracerebroventricular delivery, provide direct access to the cerebrospinal fluid but are more invasive and less suitable for repeated administration [26]. The refinement of delivery strategies will likely determine the clinical feasibility of these therapies, as bedside-compatible approaches will have a greater chance of adoption in neurology practice.

Among orthobiologics, platelet-derived preparations are gaining attention. Platelet-rich plasma and its exosome-rich derivatives contain concentrated pools of growth factors, chemokines, and vesicles with strong anti-inflammatory and pro-survival properties [27,28]. Experimental work has shown that platelet-derived extracellular vesicles delivered intranasally can attenuate neuronal loss and behavioral deficits in traumatic and PD models [29]. Since platelets can be extracted from autologous and abundant sources, they may provide a standardized and cost-effective platform for regenerative neurology, particularly if protocols for characterization and potency testing can be harmonized.

Clinical translation of orthobiologics in PD is already underway. A Phase I trial of intravenous allogeneic bone marrow-derived mesenchymal stem cells demonstrated safety and tolerability in Parkinson’s disease [30]; a subsequent Phase IIa study (ClinicalTrials.gov Identifier: NCT04506073) is investigating the safety and dose-finding of repeated infusions, with preliminary design and baseline data already published [31]. Albeit early, such trials reflect a growing acceptance of biologics as disease-modifying candidates in neurodegenerative diseases. Parallel approaches are being tested in cell-replacement therapies, where progenitors derived from stem cells can be implanted into the striatum to restore dopaminergic tone [32]. Orthobiologics occupy a complementary niche in this space, focusing less on structural replacement and more on immunomodulation, trophic support, and circuit stabilization. The two strategies may eventually synergize, with biologic signals used to support the survival and integration of grafted cells.

Peptide therapy provides another avenue of intervention. Unlike orthobiologics, which often act through complex mixtures of signals, peptides offer more precise receptor engagement while still influencing broad downstream pathways. Glucagon-like peptide-1 (GLP-1) receptor agonists have shown consistent neuroprotective and metabolic effects in both preclinical and early clinical studies of AD, improving synaptic plasticity, mitochondrial function, and insulin signaling [33]. Although a recent PD trial of exenatide did not demonstrate clear disease-modifying effects in recent trials [34], the mechanistic plausibility of GLP-1 receptor engagement remains strong, and ongoing studies with other agonists may yield different outcomes. Beyond GLP-1 analogs, novel peptides are being investigated for their ability to modulate neurodegenerative pathways. Peptides such as retro-inverse peptide inhibiting tau aggregation (RI-AG03) and tau “off-pathway” peptide (ISAD1) have shown efficacy in suppressing tau aggregation or redirecting it toward non-toxic assemblies, thereby reducing tau-mediated neurotoxicity [35,36]. In the context of PD, synthetic αSyn aggregation inhibitors, including K84s, K102s, and the CS2 decoy peptide, have been reported to block αSyn oligomerization or disrupt its pathological interactions, attenuating dopaminergic neuron injury [37,38]. Moreover, mitochondrially targeted peptides like SS-31 (Elamipretide) act by stabilizing cardiolipin in the inner mitochondrial membrane, restoring mitochondrial membrane potential, reducing reactive oxygen species (ROS), preserving synaptic proteins, and improving cognitive or behavioral outcomes in preclinical models of AD and PD [39,40,41]. These peptide approaches may also intersect with vesicle-based delivery, where encapsulation or conjugation could overcome stability and penetration barriers [42].

Taken together, the rationale for examining orthobiologics and peptides in AD and PD is compelling. Both classes of therapy are grounded in the biology of signaling and modulation rather than replacement or inhibition, addressing multiple axes of disease simultaneously, targeting inflammation, mitochondrial dysfunction, vascular integrity, and synaptic health. Both strategies are advancing toward delivery formats that could be implemented at the bedside without prohibitive invasiveness. This review synthesizes the mechanistic evidence, preclinical data, and emerging clinical findings surrounding these approaches, while also mapping the practical considerations of delivery, safety, and regulatory frameworks. The aim is not only to document the state of the science but also to outline how neurosurgeons and neurologists can begin to conceptualize regenerative protocols for neurodegeneration that extend beyond symptomatic management and toward preservation of neural circuits.

In addition to their therapeutic relevance for established disease, these modalities may also offer value in the earliest phases of neurodegeneration. Several of the dysregulated processes that precede clinical symptoms, such as low-grade neuroinflammation, mitochondrial fragility, impaired insulin signaling, and subtle synaptic injury, emerge many years before the diagnosis of AD or PD [43,44,45]. Experimental work suggests that extracellular vesicles, mesenchymal stromal cell derivatives, platelet-derived factors, GLP-1 receptor agonists, and mitochondria-targeted peptides can attenuate these early disruptions, which raises the possibility of delaying progression toward symptomatic stages [46,47]. Although still exploratory, these concepts support the broader view that orthobiologics and peptide-based interventions may hold preventive potential in the prodromal landscape of neurodegeneration.

Mild Cognitive Impairment (MCI) represents an intermediate state between normal aging and AD state and captures many of the molecular disturbances that precede dementia [48]. These include early synaptic vulnerability, impaired neurovascular regulation, low-grade inflammation, and metabolic dysfunction that converge long before overt clinical decline [49,50,51]. Several of the modalities that will be discussed in this review, including extracellular vesicles, mesenchymal stromal cell factors, and GLP-1 receptor agonists, demonstrate the capacity to influence these early biological disturbances in experimental settings. This supports the broader view that regenerative and signaling-based strategies may have relevance not only for symptomatic disease but also for prodromal conditions such as MCI.

## 2. Neurodegenerative Pathophysiology

AD and PD share a complex and overlapping pathophysiological framework. AD is defined by extracellular deposition of Aβ peptides and intracellular accumulation of hyperphosphorylated tau, leading to toxic oligomer formation, disruption of axonal transport, and synaptic dysfunction; PD is characterized primarily by misfolding and aggregation of αSyn into Lewy bodies and neurites [6,7]. Despite the different protein species, both pathologies converge on proteostatic imbalance, overwhelmed chaperone systems, impaired ubiquitin–proteasome degradation, and dysfunctional autophagy–lysosomal pathways [52,53,54]. The result is progressive accumulation of insoluble aggregates that trigger inflammatory cascades and compromise neuronal survival.

Mitochondria are central to the pathogenesis of both conditions. In AD, reduced activity of respiratory chain complexes and impaired calcium buffering create energetic deficits and oxidative stress, whereas in PD, genetic and toxin-based models consistently highlight complex I deficiency, elevated ROS, and selective vulnerability of dopaminergic neurons to mitochondrial insults [55,56]. Mitochondrial DNA mutations, defective mitophagy, and altered dynamics of fission and fusion further exacerbate neuronal vulnerability [57]. These mitochondrial impairments not only accelerate protein aggregation but also fuel glial activation, which further aggravates chronic inflammation.

Microglia and astrocytes act as both sentinels and effectors of neurodegeneration, and their sustained activation represents a shared pathophysiological hallmark. In AD, Aβ and tau oligomers stimulate innate immune pathways that drive pro-inflammatory cytokine release, complement activation, and synaptic pruning; in PD, αSyn aggregates function as danger signals that activate toll-like receptor and inflammasome signaling in microglia [58,59,60,61]. The resulting chronic inflammatory milieu perpetuates oxidative injury, impairs synaptic function, and disrupts BBB integrity [62]. Importantly, glial activation exists on a spectrum: while acute responses may be protective, sustained pro-inflammatory phenotypes dominate in late-stage disease, representing a therapeutic target for interventions that induce pro-resolving or trophic glial states [63,64].

Loss of synaptic integrity is another pivotal driver of clinical decline. Aβ oligomers in AD directly interfere with N-methyl-D-aspartate (NMDA) and alpha-amino-3-hydroxy-5-methyl-4-isooxazole-propionic acid (AMPA) receptor signaling, impair long-term potentiation, and reduce dendritic spine density, while tau pathology disrupts axonal transport and synaptic vesicle cycling [65,66]. In PD, dopaminergic denervation of the striatum impairs basal ganglia circuitry, and αSyn aggregates at presynaptic terminals compromise neurotransmitter release [67]. As these synaptic insults accumulate, network-level disconnection emerges, correlating more strongly with clinical progression than frank neuronal loss.

Neurovascular unit dysfunction adds another dimension. In AD, pericyte degeneration and endothelial failure weaken the BBB, allowing peripheral immune cell infiltration and impairing amyloid clearance across the vasculature, while in PD, vascular leakage and microvascular rarefaction are seen in the substantia nigra and striatal regions [68,69,70]. This vascular compromise combines with mitochondrial and inflammatory insults to accelerate neuronal degeneration.

Genetic and epigenetic factors further influence disease trajectories. In AD, apolipoprotein E4 (APOE4) status alters lipid metabolism, amyloid clearance, and synaptic repair; in PD, mutations in LRRK2, PARKIN, PINK1 and SNCA genes converge on mitochondrial dysfunction, impaired autophagy, and αSyn pathology [71,72,73]. Epigenetic modifications, including DNA methylation, histone acetylation, and non-coding RNA activity, shape neuroinflammatory and proteostatic networks [74], underscoring the need for interventions capable of altering signaling landscapes broadly rather than targeting single mutations.

Lastly, systemic influences also play a role. Insulin resistance and impaired glucose utilization, in particular, are prominent in AD, driving synaptic deficits and accelerating amyloid deposition, while metabolic syndrome increases risk and worsens outcomes in PD [75,76]. Moreover, altered gut microbiota, chronic low-grade systemic inflammation, and peripheral immune activation feed into central pathology, creating a whole-body contribution to vulnerability of the nervous system [77].

## 3. Orthobiologics and Peptides as Interventions in Alzheimer’s and Parkinson’s Disease

The evidence base for orthobiologic and peptide therapy for neurodegenerative diseases is still in its infancy compared with cardiovascular or oncologic biologics. However, the rapid and ongoing expansion of this field may provide a coherent rationale for continued translational development.

### 3.1. Mesenchymal Stromal Cell Derivatives

To begin with, MSC derivatives illustrate the signaling paradigm most clearly. Initial enthusiasm for cell transplantation in neurodegenerative disease has shifted toward an appreciation of the secretome and extracellular vesicles produced by these cells. These vesicles carry microRNAs (miRNAs), proteins, lipids, and mitochondrial fragments that exert anti-inflammatory, antioxidant, and pro-synaptic effects [78]. In Alzheimer’s models, administration of MSC-derived vesicles has reduced amyloid burden, attenuated tau hyperphosphorylation, and improved learning performance [78,79]. In Parkinson’s models, similar preparations have preserved dopaminergic neurons and enhanced motor function, with evidence of restored mitochondrial complex activity [80]. Importantly, these effects are not dependent on long-term engraftment of donor cells but arise from transient pulses of paracrine signaling, making the approach more compatible with practical delivery strategies. Clinical translation is underway, with early-phase trials exploring repeated intravenous or intrathecal infusions of mesenchymal derivatives in PD to establish safety and dose parameters.

### 3.2. Bone Marrow Products

Bone marrow products offer another avenue. Bone marrow aspirate and bone marrow concentrate are already popular orthobiologic approaches in regenerative medicine, and their application to neurology leverages a complex mix of progenitors, stem cells, and platelet components [81]. The secretome from bone marrow fractions contains angiogenic and neurotrophic factors that may stabilize vasculature and promote neuronal survival [82]. Previous studies have suggested that bone marrow-derived products can reduce neuroinflammation and promote synaptic recovery [83], though the clinical literature is far less developed than in musculoskeletal applications. The rationale here lies in the point-of-care feasibility: aspirated and minimally manipulated autologous products such as BMA could theoretically be administered intrathecally or intranasally without the manufacturing delays associated with culture-expanded cells, offering a rapid and safer intervention for neurodegenerative conditions.

### 3.3. Platelet-Derived Approaches

Platelet-based products extend the orthobiologic spectrum with a distinct emphasis on growth factor enrichment and vesicle content. Platelets release a rich cargo of numerous growth factors and neurotrophins, packaged within extracellular vesicles that are small enough to traverse biological barriers [84,85]. Intranasal delivery of platelet-derived vesicles has shown neuroprotective and behavioral benefits in models of traumatic brain injury and PD, highlighting the feasibility of using an abundant autologous source for central nervous system repair [29,86]. Because platelet products are already deployed widely in orthopedics, dentistry, aesthetics and dermatology, their adaptation to neurology is logistically feasible. Standardization, however, still remains challenging, as variability in platelet counts, preparation protocols, and vesicle isolation methods can influence potency [87]. Nonetheless, the combination of accessibility, safety profile, and mechanistic plausibility positions platelet-derived biologics as a pragmatic candidate for early clinical studies in neurodegenerative diseases.

### 3.4. Adipose-Derived Products

Adipose-derived orthobiologics, including stromal vascular fraction (SVF) and microfragmented adipose tissue (MFAT), provide additional signaling repertoires with strong immunomodulatory properties [88,89]. While their use in the central nervous system is still speculative, preclinical data suggest that adipose-derived stromal cells secrete exosomes rich in anti-inflammatory RNAs and factors that promote synaptic plasticity [90]. Given their relative ease of harvest, minimal invasiveness and autologous origin, these tissues represent another point-of-care option for neurology once delivery and processing hurdles are addressed.

### 3.5. Peptide Therapy

Peptide therapy complements orthobiologics by introducing precision within the signaling space. The most advanced example is the class of GLP-1 receptor agonists. Originally developed for diabetes, these peptides have shown neurotrophic, anti-inflammatory, and metabolic effects in models of both AD and PD [33,91]. They enhance insulin signaling, improve mitochondrial efficiency, reduce oxidative stress, and promote synaptic plasticity [33,91]. Clinical studies in AD are ongoing, and early signals suggest possible cognitive benefits in patients with metabolic comorbidities. In PD, the translational scenario is more complex: whereas small trials of exenatide suggested motor improvements, a large phase 3 study did not confirm disease-modifying effects [92]. The mixed results underline the importance of trial design, patient selection, and biomarker anchoring. Even if efficacy is ultimately modest, GLP-1 receptor agonists remain valuable both as monotherapies and as partners in combination strategies, particularly when paired with orthobiologic signals that converge on inflammation and mitochondrial health.

Beyond GLP-1 analogs, an expanding repertoire of neuroactive peptides is under investigation. Mitochondria-targeting peptides, such as those designed to stabilize cardiolipin or reduce ROS, have shown the ability to preserve energy metabolism and neuronal survival in Alzheimer’s models [93]. Other peptides aim to modulate kinase activity relevant to tau phosphorylation or interfere with protein aggregation. For instance, RI-AG03 blocks tau aggregation by targeting VQIVYK/VQIINK hotspots [36]; structure-based peptides have been developed to inhibit Aβ fibrillization [94]; lysine-targeting peptides prevent amyloidogenic oligomerization [95]; and SUMO1-derived peptides interfere with aggregation via SUMO-interaction motifs [96]. These agents remain largely preclinical but represent a powerful conceptual bridge between molecular specificity and system-level modulation. Nevertheless, the small size of peptides offers advantages for brain penetration, and coupling them to vesicle carriers or nanoparticle formulations may further enhance delivery.

### 3.6. Delivery Strategies

Delivery science is central to the translational feasibility of both therapies. Intranasal administration has emerged as the most practical noninvasive route, allowing direct transport from the nasal mucosa to the brain via olfactory and trigeminal pathways (Figure 2). This approach has demonstrated functional rescue in animal models across a range of biologics, including exosomes, platelet vesicles, and peptides [29,97,98,99]. Intrathecal infusion provides another option, delivering concentrated biologic payloads directly into cerebrospinal fluid, but it requires procedural expertise and carries risks that limit widespread use [100,101]. Systemic delivery may still be relevant for certain peptides or engineered vesicles designed to cross the BBB, though bioavailability remains a major limitation. A hybrid approach may work well, combining bedside-friendly intranasal administration for chronic dosing with occasional intrathecal delivery for induction or high-dose pulses.

While challenges remain in standardization, scalability, and regulatory acceptance, the foundation is already in place. By building on lessons from orthopedics and other regenerative fields, the next phase of work can begin to shape biologic protocols tailored to neurodegeneration. The central question is no longer whether such approaches are possible but how to integrate them safely, effectively, and pragmatically into neurology and neurosurgery. A synthesis of how orthobiologics and peptides intersect with major pathophysiological axes, delivery routes, and strategic considerations is presented in Table 1.

## 4. Future Directions

The enthusiasm for orthobiologics and peptide-based therapies in neurodegenerative diseases must be tempered by careful consideration of safety, feasibility, and regulatory oversight. Unlike pharmacological agents that undergo extensive research and analysis of pharmacokinetic and toxicological characterization, biologics introduce layers of complexity related to source material, preparation, dosing, and delivery route. Translating laboratory findings into pragmatic interventions for patients demands rigorous attention to these dimensions, with lessons drawn from regenerative medicine, cell therapy, and peptide pharmacology.

Safety begins with the biological source(s). MSC derivatives, whether obtained from bone marrow, adipose tissue, or umbilical cord, are generally considered safe when minimally manipulated and used autologously or in well-characterized allogeneic products [108,109]. However, risks such as contamination, immunogenicity, and tumorigenicity cannot be ignored. The shift toward extracellular vesicles and secretome has mitigated some concerns. Although these products are cell-free and lack proliferative potential, challenges still remain in defining potency, purity, and stability. BMA and PRP carry the advantage of being autologous and point-of-care, reducing risks of immune rejection, but variability in patient factors and processing protocols complicates reproducibility [110,111].

Peptide therapy, on the other hand, presents a different safety profile. Their small size and defined sequences allow for precise pharmacokinetic modeling and manufacturing control, but they are susceptible to degradation and often require chemical modification or formulation to achieve stability and cell penetration [112]. Safety signals for GLP-1 receptor agonists are generally favorable, with established results in diabetes care, but their translation into neurodegeneration requires careful monitoring for gastrointestinal side effects, weight loss, and cardiovascular effects that may confound neurological outcomes [113,114]. Novel peptides that modulate protein aggregation or mitochondrial dynamics remain at an earlier stage, and off-target effects must be anticipated, particularly when interfering with ubiquitous kinases or structural proteins.

Delivery strategies introduce additional considerations. While the intranasal route is convenient for its minimal invasiveness, it demands attention to formulation, dosing frequency and potential effects on the nasal mucosa. Chronic dosing regimens may alter local immunity or mucociliary clearance, and the long-term implications are not fully understood. Intrathecal and intracerebroventricular delivery provide direct access but carry procedural risks including infection, hemorrhage, and patient discomfort. Balancing efficacy with invasiveness will be central to the translational roadmap, and device design will play a major role in determining whether these therapies can be delivered routinely in outpatient settings or remain confined to specialized centers.

From a regulatory standpoint, orthobiologics straddle categories that complicate oversight. Autologous, minimally manipulated preparations such as BMA or PRP often fall under less stringent frameworks, though regulations vary across jurisdictions [115,116]. In contrast, culture-expanded stromal cells and engineered extracellular vesicles are generally classified as advanced therapy medicinal products, requiring rigorous preclinical data, manufacturing under good practices, and formal clinical trials [117]. Regulatory agencies such as the Food and Drug Administration (FDA), the European Medicines Agency (EMA) and the Brazilian National Health Surveillance Agency (ANVISA) have emphasized risk-based approaches that consider manipulation level, delivery route, and intended indication. For peptides, the pathway is more straightforward, aligning with conventional drug development, though combination strategies with orthobiologics may require hybrid regulatory models. The field must therefore anticipate regulatory scrutiny early, incorporating standardized assays, potency metrics, and long-term safety monitoring into study designs.

Another dimension of the translational roadmap is biomarker development. Objective markers of target engagement and disease modification are essential for moving beyond anecdotal reports and open-label case series. Imaging modalities such as PET and MRI, fluid biomarkers including Aβ, tau, αSyn, and neurofilament light, and metabolic indicators such as insulin sensitivity and mitochondrial function will need to be integrated into trial protocols [118,119]. For orthobiologics, demonstrating that extracellular vesicles reach the brain and alter inflammatory or metabolic markers is critical. For peptides, showing receptor engagement and downstream pathway modulation will validate mechanistic plausibility. Without biomarkers, even promising clinical signals risk being dismissed as placebo or regression to the mean.

Standardization also extends to trial design. Early-phase studies must prioritize safety, dose-finding, and biomarker validation, while later-phase studies should incorporate robust randomization, blinding, and clinically meaningful endpoints. Combination strategies present additional complexity, as interactions between orthobiologics and peptides may be synergistic or antagonistic depending on timing, dose, and patient characteristics. Adaptive trial designs may provide a flexible framework for testing such combinations, allowing efficient iteration without compromising rigor.

Economic and logistical factors cannot be overlooked. Autologous preparations are attractive for their safety and accessibility but may be resource-intensive and variable in potency. Allogeneic and off-the-shelf products offer scalability but demand more stringent regulation. Peptides benefit from established manufacturing pipelines but may face high costs if chronic dosing is required. Ultimately, therapies must be not only effective but also affordable and deliverable within standard healthcare infrastructures. Otherwise, they risk remaining experimental or accessible only to a small subset of patients.

### Limitations

Current evidence supporting orthobiologics and peptide-based interventions remains constrained by several hurdles. Preclinical models only partially recapitulate the complexity of human neurodegeneration, and orthobiologic preparations vary according to patient biology and processing techniques, limiting reproducibility across studies. Early clinical data are encouraging but remain limited, heterogeneous, and short in duration, with inconsistent biomarker integration. Regulatory pathways differ substantially across jurisdictions, influencing study design and clinical implementation. These limitations should be considered when interpreting the existing literature and planning biomarker-guided, standardized trials capable of demonstrating true disease modification.

The translational roadmap, therefore, involves a series of coordinated steps. At the preclinical level, rigorous characterization of products, mechanisms, and biomarkers is needed to establish a scientific foundation. At the early clinical level, safety, feasibility, and biomarker engagement must be demonstrated in small, well-controlled cohorts. At the late clinical level, larger trials must prove efficacy and reproducibility across diverse populations. Parallel to these steps, regulatory frameworks must adapt to accommodate the unique features of orthobiologics while preventing misuse, and professional societies must provide guidelines that clinicians can trust.

## 5. Conclusions

Alzheimer’s disease and Parkinson’s disease share a multifactorial pathophysiology in which protein aggregation, mitochondrial dysfunction, neuroinflammation, synaptic degeneration, and vascular compromise interact to drive progressive decline. Conventional single-target therapies have shown limited benefit, underscoring the need for strategies that modulate multiple biological processes simultaneously.

Orthobiologics and peptide-based interventions offer such a framework. Mesenchymal stromal cell derivatives, bone marrow and platelet-derived preparations, and extracellular vesicles provide paracrine signals that reprogram glia, enhance mitochondrial resilience, and support synaptic and vascular repair. In parallel, peptide therapeutics such as glucagon-like peptide-1 receptor agonists and aggregation-modulating sequences engage defined molecular pathways that complement the broader effects of orthobiologics. Together, these modalities act on mechanisms essential for circuit preservation rather than symptomatic relief alone.

Preclinical studies consistently demonstrate neuroprotective effects in both diseases, and early human trials highlight safety and feasibility. The path forward requires rigorous standardization, biomarker integration, and delivery optimization, with intranasal administration emerging as a promising route. By aligning the broad signaling capacity of orthobiologics with the precision of peptides, neurology can progress toward regenerative strategies that aim not only to slow decline but also to preserve function and improve long-term outcomes in neurodegenerative disease.

## Figures and Tables

**Table 1:** Conceptual framework for orthobiologics and peptide strategies in Alzheimer’s and Parkinson’s disease.

Pathophysiological Axis	Orthobiologic Contributions	Peptide Contributions	Delivery Options	Strategic Notes	References
Protein aggregation and proteostasis	MSC exosomes modulating autophagy and proteostasis; platelet vesicles providing protease regulators	Tau- and α-synuclein–modulating sequences; peptides blocking aggregation	Intranasal, engineered vesicle carriers	Orthobiologics act broadly, peptides add specificity to aggregation pathways	[27,96,102,103]
Mitochondrial dysfunction	Vesicle transfer of antioxidant enzymes, mitochondrial fragments; BMAC trophic factors	GLP-1 receptor agonists restoring bioenergetics; mitochondria-targeting peptides	Intranasal, IV, intrathecal	Orthobiologics stabilize redox environment, peptides fine-tune metabolic signaling	[4,40,73,82]
Neuroinflammation and glial activation	MSC vesicles reprogramming microglia; PRP exosomes reducing cytokines	GLP-1 receptor agonists attenuating inflammatory cascades	Intranasal, IV	Shared target: glial tone; synergy plausible if combined	[4,27,29,104,105]
Synaptic degeneration and network disconnection	Vesicles restoring synaptic proteins and neurotransmitter release	Peptides enhancing synaptic plasticity and receptor function	Intranasal, intrathecal	Circuit preservation is the final readout; orthobiologics supply broad cues, peptides sharpen synaptic response	[4,40,78]
Vascular and BBB compromise	Platelet-derived vesicles stabilizing endothelium, enhancing perfusion	Indirect vascular benefits from metabolic peptides	Intranasal, IV	BBB stabilization critical for delivery of both classes	[19,33,106,107]

Abbreviations: MSC, mesenchymal stromal cell; PRP, platelet-rich plasma; BMAC, bone marrow aspirate concentrate; IV, intravenous; BBB, blood–brain barrier.

## Data Availability

No new data were created or analyzed in this study.
